# HAMPLE: deciphering TF-DNA binding mechanism in different cellular environments by characterizing higher-order nucleotide dependency

**DOI:** 10.1093/bioinformatics/btad299

**Published:** 2023-05-04

**Authors:** Zixuan Wang, Shuwen Xiong, Yun Yu, Jiliu Zhou, Yongqing Zhang

**Affiliations:** School of Computer Science, Chengdu University of Information Technology, Chengdu 610225, China; School of Computer Science, Chengdu University of Information Technology, Chengdu 610225, China; School of Computer Science, Chengdu University of Information Technology, Chengdu 610225, China; School of Computer Science, Chengdu University of Information Technology, Chengdu 610225, China; School of Computer Science, Chengdu University of Information Technology, Chengdu 610225, China

## Abstract

**Motivation:**

Transcription factor (TF) binds to conservative DNA binding sites in different cellular environments and development stages by physical interaction with interdependent nucleotides. However, systematic computational characterization of the relationship between higher-order nucleotide dependency and TF-DNA binding mechanism in diverse cell types remains challenging.

**Results:**

Here, we propose a novel multi-task learning framework HAMPLE to simultaneously predict TF binding sites (TFBS) in distinct cell types by characterizing higher-order nucleotide dependencies. Specifically, HAMPLE first represents a DNA sequence through three higher-order nucleotide dependencies, including *k*-mer encoding, DNA shape and histone modification. Then, HAMPLE uses the customized gate control and the channel attention convolutional architecture to further capture cell-type-specific and cell-type-shared DNA binding motifs and epigenomic languages. Finally, HAMPLE exploits the joint loss function to optimize the TFBS prediction for different cell types in an end-to-end manner. Extensive experimental results on seven datasets demonstrate that HAMPLE significantly outperforms the state-of-the-art approaches in terms of auROC. In addition, feature importance analysis illustrates that *k*-mer encoding, DNA shape, and histone modification have predictive power for TF-DNA binding in different cellular environments and are complementary to each other. Furthermore, ablation study, and interpretable analysis validate the effectiveness of the customized gate control and the channel attention convolutional architecture in characterizing higher-order nucleotide dependencies.

**Availability and implementation:**

The source code is available at https://github.com/ZhangLab312/Hample.

## 1 Introduction

Transcription factors (TFs) play a crucial role in the gene transcription regulation program by binding to degenerate DNA sequences at specific genomic locations (Vierstra et al. 2020). These DNA sequences, known as TF binding sites (TFBSs), include some inherent characteristics: (i) higher-order nucleotide dependencies. There are physical and chemical interactions between each nucleotide and its neighboring nucleotides caused by the local three-dimension structure of DNA and the nucleotide-amino-acid contact (Wang et al. 2022); and (ii) cross-cell type reuse. Only a few TFs are engaged in the regulation program of many cell types; thus, each TF is often reused in different cell types and development stages (Zhang et al. 2023). These observations naturally raise the research question of the relationship between the cell-type-specific and cell-type-shared higher-order nucleotide dependencies and the TF-DNA binding mechanism. Therefore, predicting unknown TFBSs in diverse cell types and development stages has become a fundamental problem in bioinformatics.

Recent deep learning approaches attempt to characterize high-order dependencies among nucleotides by using novel DNA encoding strategies or feeding abundant features which span multiple nucleotides. For instance, HOCNN (Zhang et al. 2019) combines the higher-order genomic sequence encoding, i.e. *k*-mer encoding, with the multi-scale CNN architecture to simultaneously capture multi-scale sequence motifs and high-order nucleotide dependencies in motifs. DLBSS (Zhang et al. 2021a) and CRPTS (Wang et al. 2021) fuse DNA sequence and shape features into the shared deep learning architecture to consider local structural information of DNA sequences, where DNA shape features produced by modeling physical interactions between neighboring base-pairs encode the dependencies between nucleotides. BHSite (Zhang et al. 2021b) and CNN_TF (Zhou et al. 2020) separately learn low-order and higher-order nucleotide dependencies from DNA sequence and histone modification features by the dual-branch deep learning architecture, where the histone modification features are the epigenomic modification level of histone on chromatin structure and span several base-pairs. Subsequently, variants of these approaches are put forward one after another (Zhou et al. 2019; Zhang et al. 2022a,b,d). However, current deep learning approaches should improve in some respects: (i) feature combination. Leveraging *k*-mer encoding, DNA shape, and histone modification features simultaneously can better characterize high-order nucleotide dependencies; (ii) model architecture. Designing an architecture to adaptively capture cell-type-specific and cell-type-shared high-order nucleotide dependencies for predicting TFBS in difference cellular environments remains challenging; and (iii) model interpretation. Interpreting what biological features learned by deep learning models is necessary to analyze TF-DNA binding mechanism.

Here, we propose a multi-task learning framework HAMPLE to simultaneously predict TF-DNA binding events in different cell types by jointly integrating the *k*-mer encoding, DNA shape, and histone modification features. First, HAMPLE utilizes the higher-order nucleotide dependency embedding to represent a DNA sequence based on the *k*-mer encoding, DNA shape, and histone modification features. Then, HAMPLE separately extracts cell-type-specific and cell-type-shared DNA binding motifs and epigenome languages from three types of higher-order nucleotide dependencies by the shared and specific expert modules in the customized gate control model. For a particular cell type, the corresponding TFBS prediction module absorbs biological knowledge from the shared and specific expert modules to predict whether the current TF can bind a given DNA sequence. Finally, HAMPLE leverages the joint loss optimization to coordinatively train the shared and specific expert modules in an end-to-end manner. To automatically capture crucial biological features associated with TF-DNA binding and suppress unnecessary ones, HAMPLE uses the convolutional block based on channel mechanism (Li et al. 2022) as the fundamental block of the customized gate control model (Tang et al. 2020) to perform feature extraction and introduces the gating module to combine shared and specific features. Notably, the convolutional block and gating module are easily interpretable.

To verify the effectiveness of our proposed HAMPLE framework, we conduct extensive experiments on seven public TFBS datasets. Experimental results demonstrate that HAMPLE yields better auROC performance than several state-of-the-art approaches. Importance analysis of each higher-order nucleotide dependency pinpoints that *k*-mer encoding, DNA shape, and histone modification have complementary predictive power for TF-DNA binding in different cellular environments. Ablation and interpretable study expound that the customized gate control model and the channel attention mechanism-based convolutional block effectively capture the biological feature from the higher-order nucleotide dependency for improving the prediction performance.

## 2 Materials and methods

### 2.1 Date collection

We download ChIP-seq datasets of transcription factors EZH2, GABPA, JUND, MAX, NRF1, RFX5, and TAF1 and of histone modifications H3K27ac, H3K36me3, H3K4me2, H3K9ac, H3K27me3, H3K4me1, H3K4me3, and H3K9me3 for cell lines GM12878, H1, HeLa-S3, HepG2, and K562 from the ENCODE database. Briefly, the ChIP-seq dataset of each TF comprises a large number of raw reads (bed files) of all binding sites of the TF in a particular cell line. The ChIP-seq dataset of each histone modification contains genome-wide signal coverage tracks (bigwig files) of the epigenomic modification level of the histone on chromatin structure. Besides, we compute DNA shape features, including Minor Groove Width (MGW), Propeller Twist (ProT), Helix Twist (HelT), and Roll, based on DNAshapeR software (Chiu et al. 2016) which uses a five-base-pair sliding window approach to calculate the double helix DNA structural features unique to each of the 512 distinct pentamers.

We pre-process ChIP-seq datasets of TFs and histone modifications and DNA shape features separately: (i) ChIP-seq datasets of TFs. By Pybedtools software (Dale et al. 2011), we center ChIP-seq binding events on 101 bp DNA sequences. (ii) ChIP-seq datasets of histone modifications. According to the chromatin coordinate of the DNA sequences, we extract the signal values of the corresponding coordinates from histone modification coverage tracks. (iii) DNA shape features. Given a DNA sequence of length *n*, we can obtain *n*-4 MGW and ProT values and *n*-3 HelT and Roll values by DNAshapeR software. To get the shape features of the same length as their corresponding DNA sequence, we had two “0” on both sides of the shape features. We took the average over the two neighboring values of HelT and Roll.

Let $\Lambda ={\left\{{\tilde{x}}_{i},{\tilde{y}}_{i}\right\}}_{i=1}^{N}$ denotes the dataset of a particular TF, where ${\tilde{x}}_{i}$ and ${\tilde{y}}_{i}$ respectively indicate the *i*th TFBS sample and corresponding label in the dataset, and *N* refers to the number of the sample. Specifically, ${\tilde{x}}_{i}$ includes the collected DNA sequence, DNA shape and histone modification; ${\tilde{y}}_{i}={\left\{{\hat{y}}_{i,k}\right\}}_{k=1}^{C}$ where ${\hat{y}}_{i,k}\in \{0,1\}$ indicates whether the particular TF binds the *i*th TFBS sample in the *k*th cell line, and C refers to the number of the cell line. For dataset Λ, 60% samples are used as the TrainingSet, 20% as the ValidationSet, and 20% as the TestingSet (Supplementary Table S1).

### 2.2 Overall framework of HAMPLE

**Figure 1.**
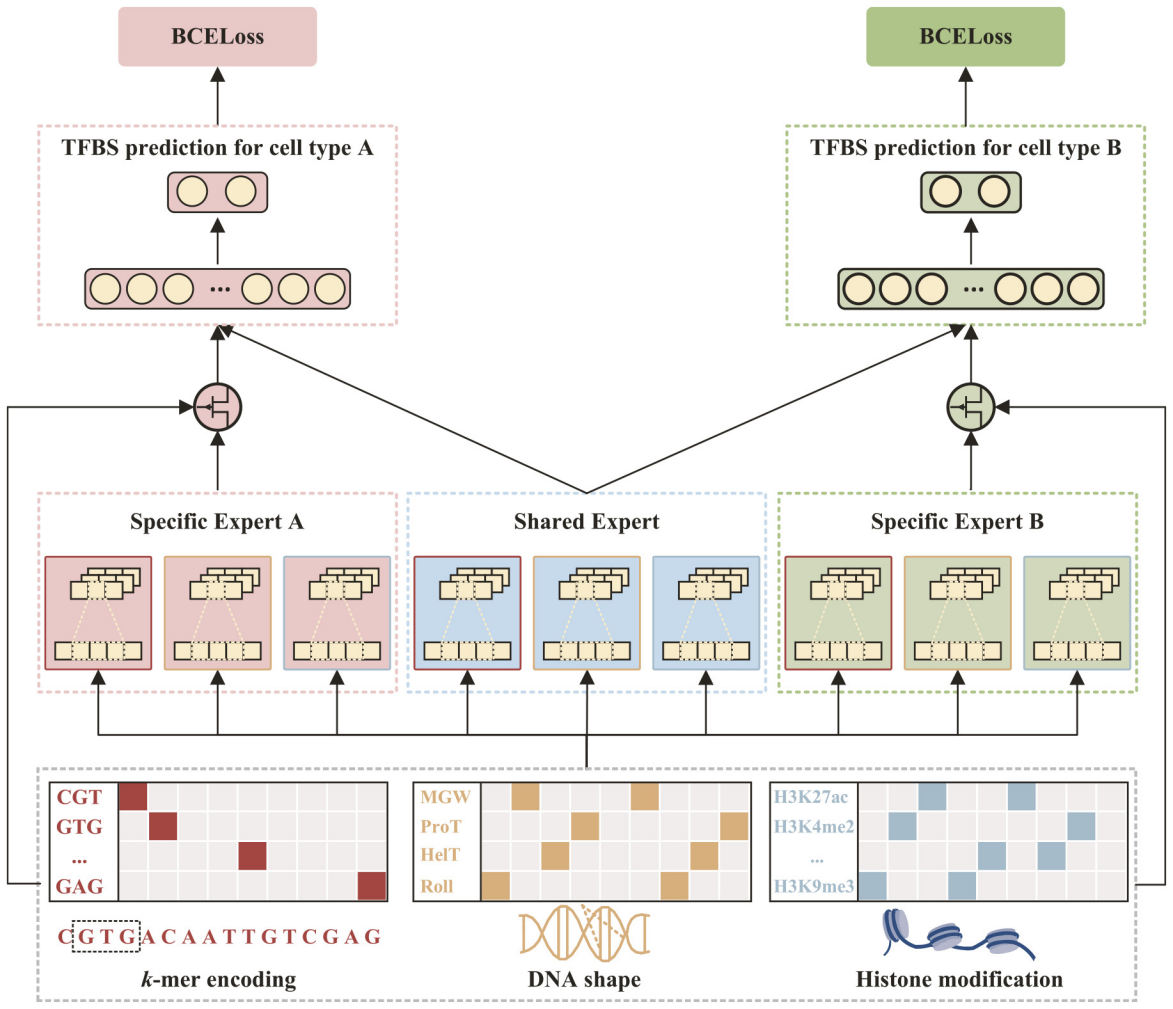
Overall framework of HAMPLE.

The overall framework of HAMPLE comprises the higher-order nucleotide dependency embedding, the customized gate control model, and the joint loss optimization (Fig. 1). Specifically, the goal of the higher-order nucleotide dependency embedding is to characterize the DNA sequence by *k*-mer encoding, DNA shape, and histone modification features and map three types of features into the latent space with the same dimension; the customized gate control model first uses the shared and specific biological expert modules to extract cell-type-shared and cell-type-specific DNA binding motifs or epigenomic languages from the higher-order nucleotide dependency features where the convolutional block based on the channel attention mechanism is regarded as the basic block of each expert module and then exploits the several TFBS prediction modules to predict whether a given DNA sequence can be bound by the TF in the corresponding cell type; the joint loss optimization aims to coordinatively optimize shared and specific biological expert modules by the weighted sum of the losses for each TFBS prediction.

### 2.3 Higher-order nucleotide dependency embedding

Three types of higher-order nucleotide dependencies are used to represent a DNA sequence: *k*-mer encoding, DNA shape, and histone modification features. Given a DNA *L*-bp sequence $\tilde{S}=({\tilde{s}}_{1},\ldots ,{\tilde{s}}_{i},\ldots {\tilde{s}}_{L})$, *k*-mer encoding describes the given DNA sequence as a non-independent feature matrix *K* of size $4^{h}\times L$, where *h* indicates the degree of the order. This is an example for 2-order encoding: AA = [1, 0, 0, 0, 0, 0, 0, 0, 0, 0, 0, 0, 0, 0, 0, 0], AC = [0, 1, 0, 0, 0, 0, 0, 0, 0, 0, 0, 0, 0, 0, 0]. The DNA shape features are directly signified as a feature matrix *S* of size 4×L: where $s_{i}$ indicates the *i*th shape vector of size 4×1. Based on the previous study (Zhou et al. 2020), we use the following scheme to generate the histone modification feature matrix: we first compute the histone modification features for all non-overlapping 25 bp bins and then calculate the histone modification features for each 100 bp bins by averaging four 25 bp bins within it. Finally, we concatenate the histone modification features of the *L* 100-bp bins to generate the feature matrix of size 8×L: where $h_{i}$ indicates the *i*th histone modification vector of size 8×1. After feature embedding, we exploit three convolutional layers (kernel size = 1) to map the three feature matrices into a latent space with the same dimension of 4×L.


(1) $$S=(s_{1},s_{2},\ldots ,s_{i},\ldots ,s_{L-1},s_{L})$$

(2) $$H=(h_{1},h_{2},\ldots ,h_{i},\ldots ,h_{L-1},h_{L})$$

### 2.4 Convolutional block based on channel attention mechanism

Given a feature map *x* which comprises any high-order nucleotide dependence information in *k*-mer encoding, DNA shape or histone modification, we use a convolutional layer (kernel size = motif length of current TF) to capture TF-DNA binding motifs or epigenomic languages from high-order nucleotide dependence information: where ReLU(⋅) defines the ReLU function and ${C1D}_{m}(\cdot )$ defines the one-dimensional convolution with the kernel size of *m*. *m* is the motif length of the current TF. $\tilde{x}$ indicates the output feature map of the convolutional layer.


(3) $$\tilde{x}=\text{ReLU}({C1D}_{m}(x))$$

**Figure 2.**
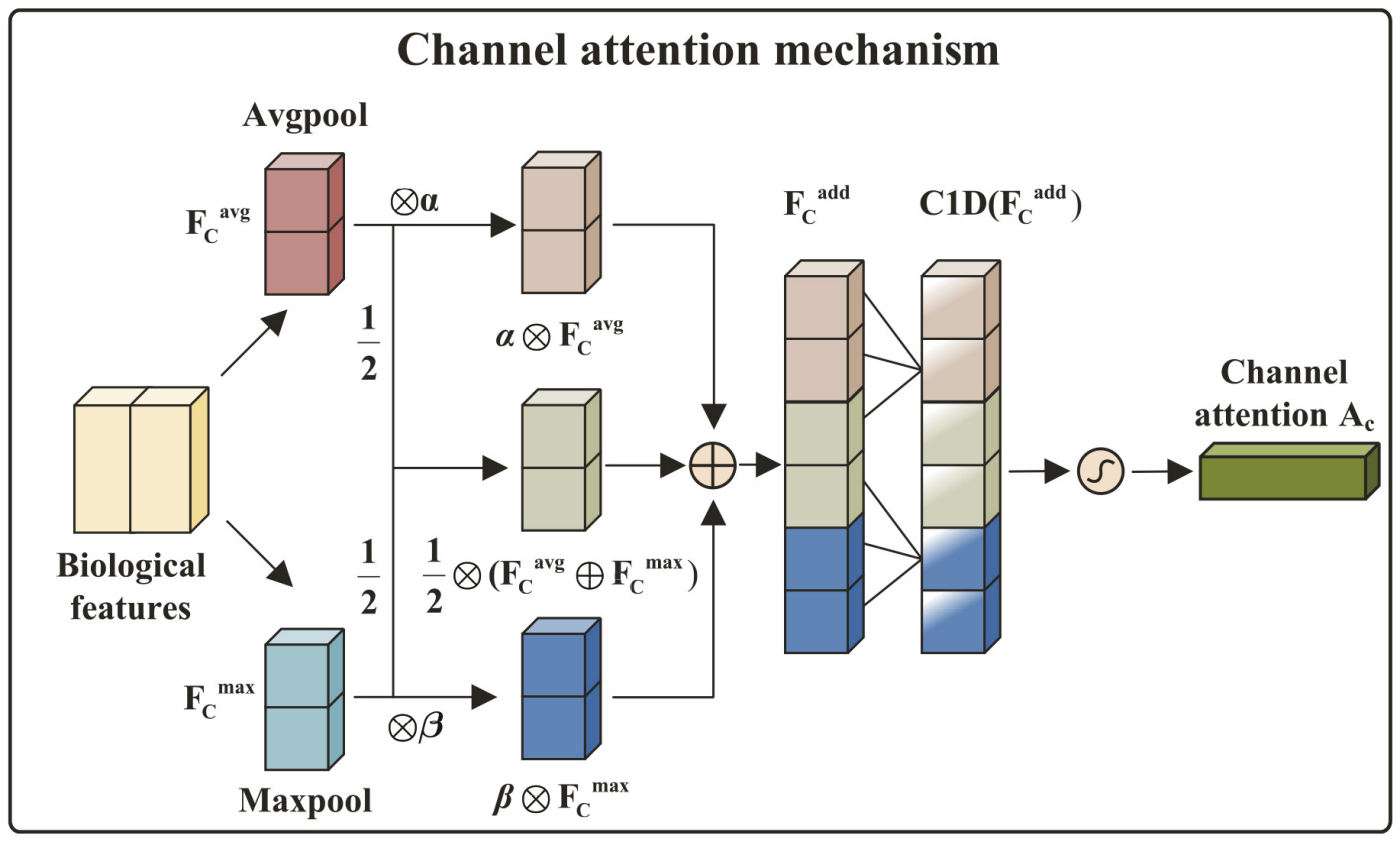
Diagram of channel attention mechanism.

Each kernel (channel) in the convolutional layer represents a DNA binding motif or epigenomic language derived from the corresponding high-order nucleotide dependence information. However, DNA binding processes of a particular TF in different cell types are driven by distinct combinations of motifs or epigenomic languages. Thus, we use the channel attention mechanism to help the proposed model concentrate on kernels with crucial biological features (Fig. 2).

Based on the previous study (Li et al. 2022), we first simultaneously use the average-pooling and max-pooling to aggregate the context information of the feature map $\tilde{x}$ and then exploit an adaptive mechanism in average-pooled features and max-pooled features to generate the channel descriptor which characterizes the importance of each biological feature to current TF-DNA binding process: where GlobalAvgPool1D(⋅) and GlobalMaxPool1D(⋅) define the one-dimensional global average-pooling and global max-pooling, respectively. ⊗ and ⊕ define the element-wise multiplication and summation. α∈[0, 1] and β∈[0, 1] are the learnable parameters for the adaptive mechanism between average and max-pooled features.


(4) $$F_{c}^{\text{avg}}(\tilde{x})=\text{GlobalAvgPool}1D(\tilde{x})$$

(5) $$F_{c}^{\text{max}}(\tilde{x})=\text{GlobalMaxPool}1D(\tilde{x})$$

(6) $$\begin{array}{c} F_{c}^{\text{add}}(\tilde{x})=\alpha ⊗F_{c}^{\text{avg}}(\tilde{x})⊕\frac{1}{2}⊗(F_{c}^{\text{avg}}(\tilde{x})\\ ⊕F_{c}^{\text{max}}(\tilde{x}))⊕\beta ⊗F_{c}^{\text{max}}(\tilde{x}) \end{array}$$

To further generate the channel attention map based on the channel descriptor, we first use a fast one-dimensional convolution layer (kernel size = κ) to capture the cross-channel interaction for characterizing the relationship between different biological features and then exploit the sigmoid function to project each value in the channel attention map into [0, 1]: where $|\cdot {|}_{\text{odd}}$ defines the operation to compute the nearest odd number, σ(⋅) defines the sigmoid function, and ${C1D}_{\kappa }(\cdot )$ defines the one-dimensional convolution with kernel size of κ. κ is a parameter and indicates the interaction between κ neighbors. *C* denotes the channel number of the feature map $\tilde{x}$. γ and *b* are hyperparameters and we set them to 2 and 1, respectively.


(7) $$\kappa =|\frac{{\;\text{log}\;}_{2}(C)}{\gamma }+\frac{b}{\gamma }{|}_{\text{odd}}$$

(8) $$A_{c}(\tilde{x})=\sigma ({C1D}_{\kappa }(F_{c}^{\text{add}}(\tilde{x})))$$

Finally, we recalibrate the feature map $\tilde{x}$ based on the element-wise multiplication between the channel attention map: where ${\tilde{x}}^{\prime }$ indicates the recalibrated feature map, which emphasizes the crucial features of the TF-DNA binding motif or epigenomic language and suppresses unnecessary ones.


(9) $${\tilde{x}}^{\prime }=A_{c}(\tilde{x})⊗F(\tilde{x})$$

### 2.5 Customized gate control model

To explicitly leverage cell-type-shared and cell-type-specific high-order-nucleotide dependencies, we use a customized gate control model which comprises a series of biological expert modules at the bottom and some TFBS prediction modules at the top. Specifically, specific biological expert modules are responsible for learning DNA binding motifs and epigenomic languages of current TF in a particular cell type, while a shared biological expert module captures shared ones driven by the cross-cell type reuse phenomenon; TFBS prediction modules are in charge of inferring the probability of whether a given DNA sequence can be bound by current TF in the different cellular environment.

Each biological expert module uses three convolutional blocks based on the channel attention mechanism as the sub-expert modules to extract corresponding biological features from the *k*-mer encoding, DNA shape and histone modification, respectively. After feature extraction, we selectively combine features derived from specific and shared biological expert modules through a gating module to consider the differences and relationships between cell-type-shared and cell-type-specific high-order nucleotide dependencies (Fig. 1c). Precisely, the gating module first concatenates the extracted biological features from the specific and shared expert modules as the selected matrix, then exploits the single feed-forward layer with the max-pooling and the softmax function to learn the importance of each convolutional block in both specific and shared expert modules based on the original *k*-mer encoding, DNA shape, and histone modification; and finally calculates the weighted sum of the selected matrix: where ${\text{Linear}}_{k}(\cdot )$ defines the feed forward layer of the *k*th gating module and Softmax(⋅) defines the softmax function. ${\tilde{x}}_{(k,1),}^{\prime }$, ${\tilde{x}}_{(k,2)\;}^{\prime }$, and ${\tilde{x}}_{\left(k,3\right)}^{\prime }$ indicate the extracted biological features from three convolutional blocks in the *k*th specific expert module, ${\tilde{x}}_{(s,1),}^{\prime }$, ${\tilde{x}}_{\left(s,2\right)}^{\prime }$, and ${\tilde{x}}_{\left(s,3\right)}^{\prime }$ denote the extracted biological features from three convolutional blocks in the shared expert module, $E_{k}$ refers to the selected vector of the *k*th gating module, and $g_{k}$ is the output of the *k*th gating module.


(10) $$E_{k}=[{\tilde{x}}_{(k,1),}^{\prime }{\tilde{x}}_{(k,2),}^{\prime }{\tilde{x}}_{(k,3),}^{\prime }{\tilde{x}}_{(s,1),}^{\prime }{\tilde{x}}_{(s,2),}^{\prime }{\tilde{x}}_{\left(s,3\right)}^{\prime }]$$

(11) $$\begin{array}{c} g_{k}=\text{Softmax}({\text{Linear}}_{k}(\text{GlobalMaxPool}([K,S,H])))E_{k} \end{array}$$

Finally, each TFBS prediction module absorbs biological features from the shared expert module and its specific expert module and predicts the probability of whether a given DNA sequence can be bound by current TF in the corresponding cell type through the multi-layer perceptron with the sigmoid function: where ${\text{MLP}}_{k}(\cdot )$ defines the multi-layer perceptron of the *k*th TFBS prediction module and $y_{k}$ indicates the probability of whether a given DNA sequence can be bound by current TF in the *k*th cell type.


(12) $$y_{k}=\sigma ({\text{MLP}}_{k}(g_{k}))$$

### 2.6 Joint loss optimization

To jointly train specific and shared biological expert modules in an end-to-end manner, we perform the weighted sum of the losses for each prediction: where BECLoss(⋅) defines the binary cross entropy loss function, $\Theta_{S}$ indicates the learnable parameters of the shared biological expert module and $\Theta_{K}$ denotes the learnable parameters of the *k*th specific biological expert module. To optimize the loss function, we exploit the Adam algorithm, a universal solver for optimizing the neural network models.


(13) $$L(\Theta_{1},\ldots ,\Theta_{K},\Theta_{S})=\sum_{k=1}^{K}\text{BCELoss}(y_{k,}{\hat{y}}_{k})$$

## 3 Result

### 3.1 Overall performance of HAMPLE in TFBS prediction

**Figure 3.**
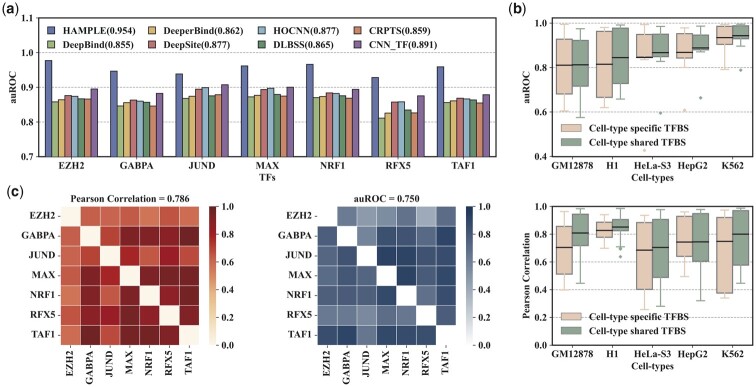
The overall performance of the proposed approach. (a) auROC performance comparison of HAMPLE with several state-of-the-art TFBS prediction models. These competitors aim to discriminate binding sites from non-binding sites of a particular TF. (b) auROC performance of HAMPLE on cell-type-shared and cell-type-specific TFBSs in the five cell types (top). Pearson correlation of cell-type-shared and cell-type-specific TFBSs in the five cell types (bottom). (c) auROC performance of cross-factor prediction of HAMPLE in the same cellular environment (left). Pearson correlation of binding sites between each pair-wise TF (right).

To explore the effectiveness of the proposed approach on TFBS prediction, we compute the auROC of HAMPLE and different TFBS prediction models, including DeepBind, DeeperBind, DeepSite, HOCNN, DLBSS, CRPTS, and CNN_TF. These competitors are well-verified models in discriminating TFBSs from non-TFBSs. To make a fair comparison, we use the same data pre-processing pipeline as the original publications. HAMPLE can outperform all competitors significantly (Fig. 3a), which reveals that the adaptive integration of cell-type-specific and cell-type-shared *k*-mer features, DNA shape features and histone modification features can better express the process of TF-DNA binding. It is noteworthy that the task of HAMPLE is more complex than other models because the specificities of TFBSs and non-TFBSs are often different. In contrast, the particularities of binding sites of the same TF in different cells are often similar. These results confirm that HAMPLE architecture advances the prediction accuracy of TFBSs in different cell types.

To validate whether the proposed approach can capture the shared high-order nucleotide dependencies of particular TFBS in different cell types, we evaluate the performance of HAMPLE on cell-type specific TFBSs and cell-type-share TFBSs, separately. The cell-type-shared TFBS of a particular cell line is defined as the TFBS with at least one similar TFBS (>75% nucleotides are consistent) in the other four cell types. The remaining TFBSs are referred to as cell-type-specific TFBSs. In addition, we compute the Pearson correlations among the five cell types for both specific and shared binding sites of each TF. HAMPLE achieves higher auROC on shared TFBSs than specific TFBSs for the five cell types (Fig. 3b, top), which reveals that the shared TFBSs dominate the performance of HAMPLE. Pearson correlations of shared TFBSs are higher than that of specific TFBSs in the five cell types (Fig. 3b, bottom), which indicates that shared TFBSs indeed have similar high-order nucleotide dependencies and explains why HAMPLE achieves higher performance for shared TFBSs than that for specific TFBSs. These results confirm that HAMPLE can effectively leverage the cross-cell-type reused TFBSs to learn the shared high-order nucleotide dependencies to improve TFBS prediction performance.

To further study different TF-DNA binding processes in the same cellular environment, we evaluate the cross-factor prediction performance of the proposed approach. Specifically, we use the HAMPLE model trained on one of seven datasets to test the remaining six datasets in rotation and compute the Pearson correlations between all datasets. In most cases, HAMPLE performs well for cross-factor prediction. However, the Pearson correlations are slightly higher (Fig. 3c), which reveals that HAMPLE may learn similar patterns of higher-order nucleotide dependencies existing between different TFBSs in the same cellular environment. These results demonstrate that HAMPLE has a good generalization ability for cross-factor prediction.

### 3.2 Importance analysis of each higher-order nucleotide dependency

**Figure 4.**
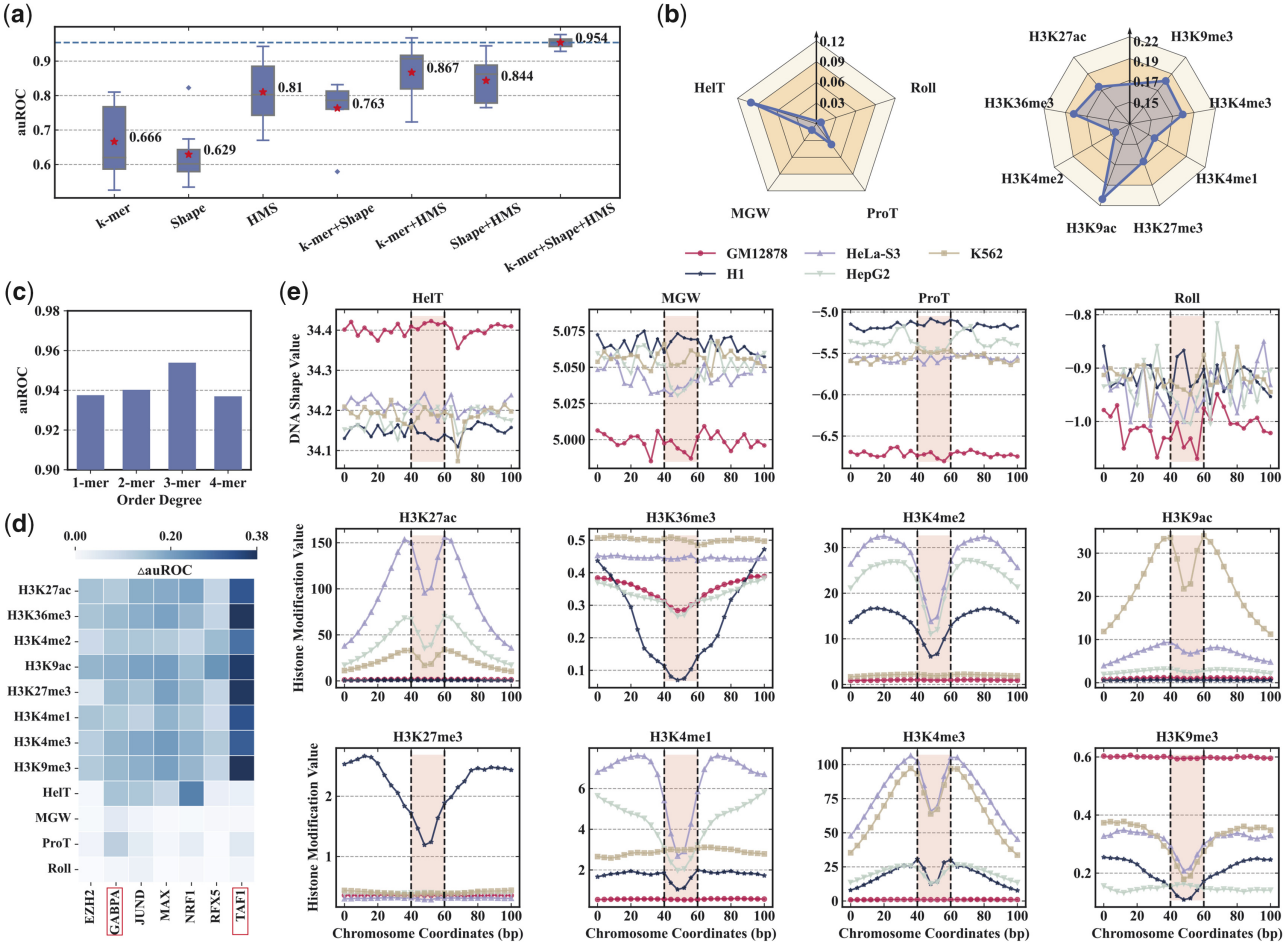
The importance analysis of each higher-order nucleotide dependency. (a) auROC performance of different combinations of higher-order nucleotide dependencies. (b) Contribution of each DNA shape relative to all four DNA shapes and each histone modification feature relative to all eight histone modifications. The contribution is measured by △auROC (the performance change of auROC). (c) auROC performance of different order degrees of the *k*-mer encoding. (d) Contribution of each DNA shape and histone modification on the binding site prediction of different TFs. (e) Visualization of the DNA shape distribution of GABPA and the histone modification distribution of TAF1 across different cell types.

We begin by evaluating the performance of combining *k*-mer encoding, DNA shape, and histone modification features in the proposed model and exploring the effect of different higher-order nucleotide dependency combinations on TFBS prediction. The *k*-mer + shape + HMS achieves the best auROC performance (Fig. 4a), which reveals that biological features extracted from the different types of higher-order nucleotide dependencies are supplementary to each other and helpful in improving the prediction performance. Specifically, the average auROC performance of *k*-mer + shape + HMS compared to that of *k*-mer, shape, HMS, *k*-mer + shape, *k*-mer + HMS, and shape + HMS increases 0.288, 0.325, 0.144, 0.191, 0.087, and 0.11, respectively.

To further investigate the contribution of each DNA shape feature relative to all four DNA shape features and each histone modification feature close to all eight histone modification features together, we conduct the leave-one-feature-out feature selection experiments to achieve that. Specifically, we train the HAMPLE framework using three DNA shape features and compare the △auROC (the performance change of auROC) performance with the HAMPLE framework trained with all four DNA shape features; the histone modification features are the same. HelT and ProT have more predictive power than the other shape features (Fig. 4b, left), which reveals that the twist of double helix DNA is the essential factor of DNA shape features to discriminate binding sites of a particular TF in the different cellular environment. The H3K9ac is the most crucial histone modification feature, and H3K36me3 is the second one (Fig. 4b, right). The function of H3K9ac is using the cell cycle to maintain the chromatin states associated with the cell-type-specific gene expression (Halsall et al. 2021). The function of H3K36me3 is regulating the gene expression of human cancer cell lines (Zhang et al. 2018). Hence, the significance of histone modification features is consistent with their biological functions. Additionally, we analyze the effect of the order degree of the *k*-mer encoding by separately evaluating the auROC performance of HAMPLE based on 1-mer, 2-mer, 3-mer, and 4-mer encoding. With the increase of the order degree, the auROC performance improves firstly and then it starts to deteriorate (Fig. 4c), which reveals that the more significant order degree leads to better representation capability, while it results in over-fitting when the order degree is too large. These results confirm the effectiveness of each type of higher-order nucleotide dependency.

**Figure 5.**
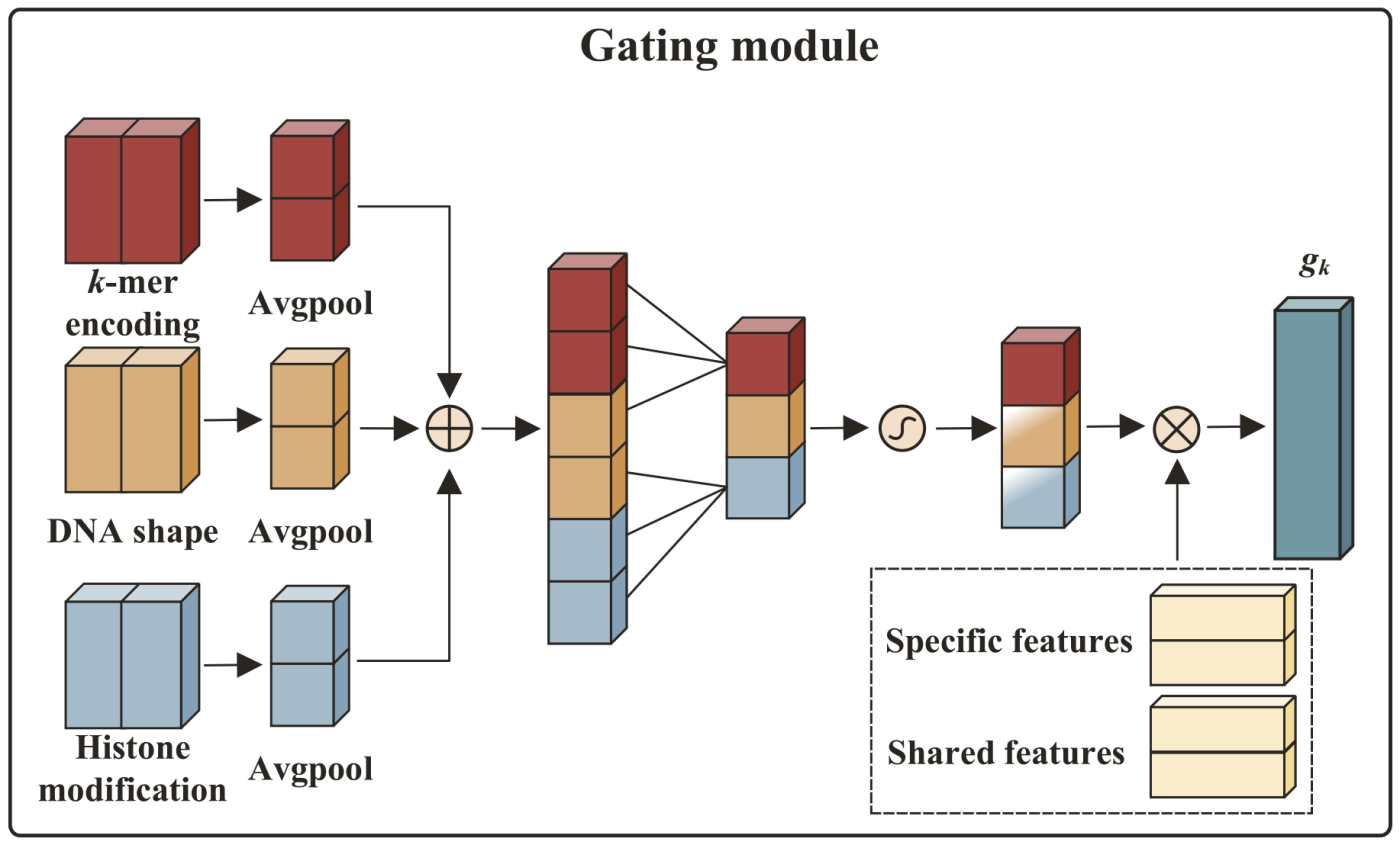
Diagram of gating module.

We finally explore the data distribution of each DNA shape and histone modification feature around TFBSs in the different cellular environments. We mainly focus on the data of the transcription factors GABPA and TAF1, where GABPA and TAF1 are the transcription factors most affected by the DNA shape and histone modification features (Fig. 4d). The data distribution of four DNA shapes displays the significant numerical difference in the GM12878 cell lines compared with the other four cell lines (Fig. 5e, top), which reveals that DNA shape features improve the prediction performance by helping the proposed framework discriminate the TFBS in the GM12878 cell lines from that in the other four cell lines. The data distribution of H3K27ac, H3K4me2, H3K9ac, H3K4me1, H3K4me3, and H3K9me3 display bimodal patterns with the numerical difference in HeLa-S3, HepG2, and K562 cell lines, while that of H3K36me3 and H3K27me3 have weak signals in the core binding sites of the H1 cell line (Fig. 4e, bottom), which indicates that histone modifications help predict TFBS in the H1, HeLa-S3, HepG2, and K562 cell lines by complex epigenomic languages. These results confirm that DNA shape and histone modification features effectively recognize binding sites of the same TF in different cellular environments.

### 3.3 Ablation and interpretable study of customized gate control and channel attention convolutional architecture

**Figure 6.**
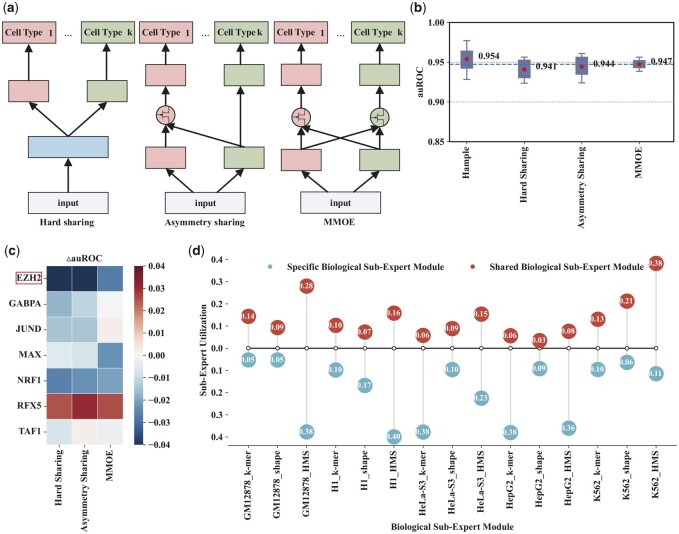
The ablation study and interpretable analysis of customized gate control. (a) Architecture of different multi-task learning models. (b) auROC performance comparison of customized gate control with different multi-task learning models. (c) △auROC between customized gate control and different multi-task learning models. (d) Utilization of different biological sub-expert modules.

To investigate why the customized gate control architecture is effective in predicting TFBS in different cellular environments, we conduct extensive ablation studies and interpretable analyses. Firstly, we analyze the utility of the customized gate control architecture by replacing it with hard sharing, asymmetry sharing and multi-gate mixture-of-experts (MMOE) architectures (Fig. 6a). The customized gate control architecture yields better auROC performance than several state-of-the-art architectures (Fig. 6b), which reveals the consideration of differences and relationships between cell-type-shared and cell-type specific higher-order nucleotide dependencies features is helpful to predict TFBS in different cellular environments. Secondly, we disclose how gating modules aggregate the shared and specific features by investigating the utilization of all sub-expert modules. We mainly focus on the data of transcription factor EZH2, which is most affected by the customized gate control architecture (Fig. 6c). The utilization between shared and specific sub-expert modules characterizing *k*-mer encoding and histone modification are significantly different, while that characterizing DNA shape are nearly similar (Fig. 6d), which indicates that the well-designed architecture of customized gate control can help achieve better differentiation between shared and specific *k*-mer encoding and histone modification features. These results confirm that the customized gate control can improve TFBS prediction performance by modeling differences and relationships between shared and specific *k*-mer encoding and histone modification features for different cellular environments.

**Figure 7.**
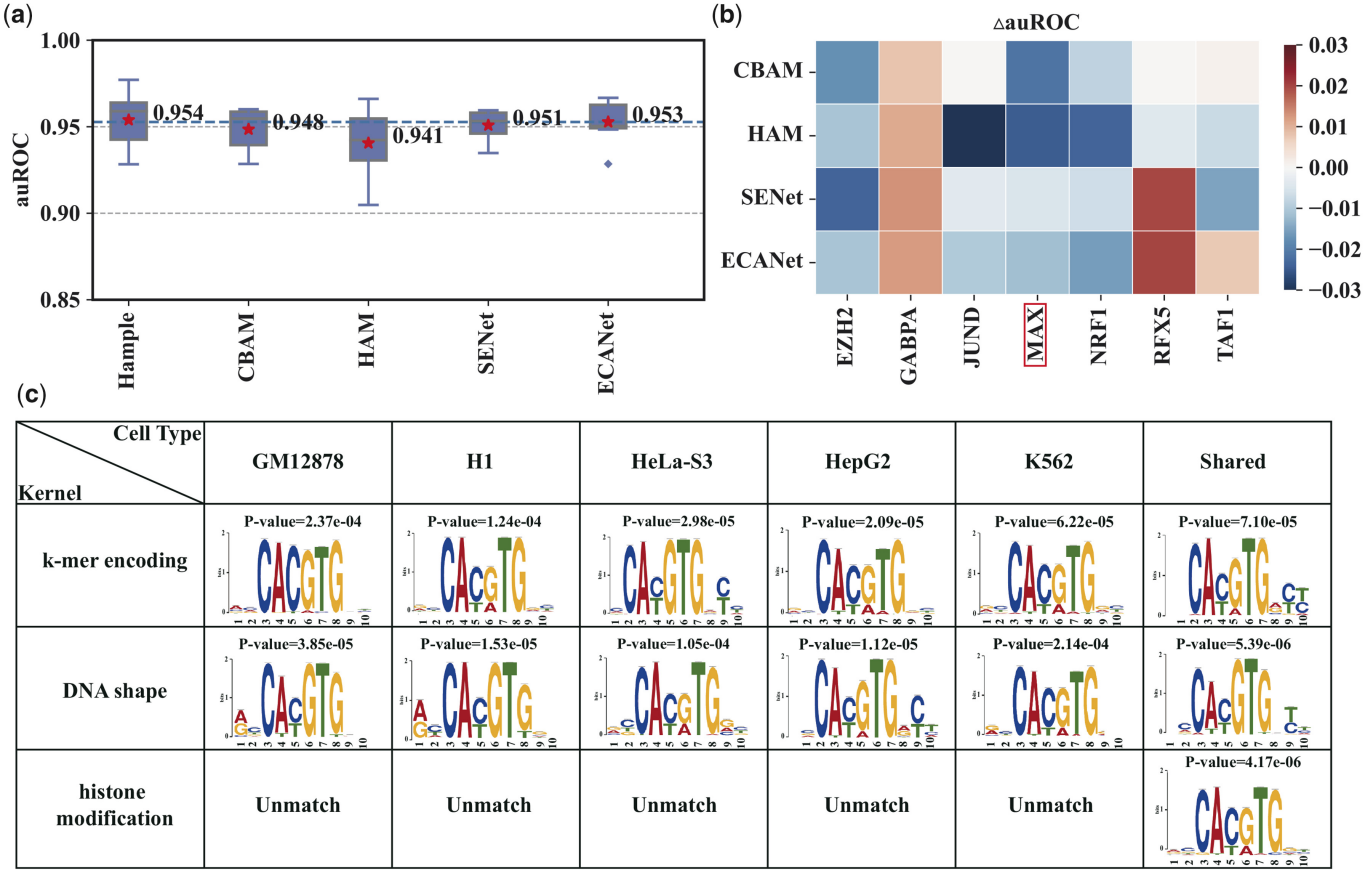
The ablation study and interpretable analysis of channel attention convolutional architecture. (a) auROC performance of our proposed channel attention mechanism with different well-known attention mechanisms. (b) △auROC between our proposed channel attention mechanism and different well-known attention mechanisms. (c) Sequence logos of TF-DNA binding motifs extracted by the convolutional kernels with the highest attention score.

Furthermore, we explore why the convolutional block based on the channel attention mechanism can improve the performance of predicting TFBS in different cellular environments. Firstly, we analyze the utility of the proposed channel attention mechanism by replacing it with CBAM (Woo et al. 2018), HAM (Li et al. 2022), SENet (Hu et al. 2020), and ECANet (Wang et al. 2020). CBAM and HAM are well-known hybrid attention mechanisms, i.e. spatial + channel attention mechanisms and SENet and ECANet are widely known channel attention mechanisms. The auROC performance of the proposed channel attention mechanism outperforms all the competitors significantly (Fig. 7a), which reveals that the spatial attention mechanism is unsuitable for TFBS prediction and the adaptive mechanism in the proposed channel attention mechanism can leverage the complementary relationship between the average-pooled and max-pooled features to enrich the biological feature map. Secondly, we explore the ability of the convolutional layer in each sub-expert module to represent the TF-DNA binding motif by extracting motifs from the convolutional kernels with the highest attention score. We mainly focus on the data of transcription factor MAX, which is most affected by the proposed attention mechanism and has the known DNA binding motif (Fig. 7b). The motifs extracted from convolutional kernels characterizing *k*-mer encoding and DNA shape can match the known motif. In contrast, convolutional kernels indicating histone modification can hardly achieve matching (Fig. 7c), demonstrating that channel attention convolutional architecture can capture sequence and shape motifs to improve the prediction performance in different cellular environments. How epigenomic languages are learned now awaits to be further investigation.

## 4 Discussion

In this article, we develop a multi-task learning framework HAMPLE to model the relationship between higher-order nucleotide dependency and TF-DNA binding mechanism across cell types. Compared with the previous studies, HAMPLE has three unique advantages: (i) it combines *k*-mer encoding, DNA shape, and histone modification features to characterize different types of higher-order nucleotide dependencies comprehensively; (ii) it simultaneously predicts binding sites of a particular TF in different cellular environments by multi-task learning; and (iii) it automatically learns cell-type-specific, and cell-type-shared features from the higher-order nucleotide dependency and significantly improves the interpretability based on the customized gate control and the channel attention mechanism. Experimental results on several TFBS datasets demonstrate that HAMPLE substantially outperforms the state-of-the-art prediction approaches by learning cell-type-specific and cell-type-shared features from the higher-order nucleotide dependencies. Moreover, we analyze different higher-order nucleotide dependencies and find that *k*-mer encoding, DNA shapes, and histone modifications have strong predictive power for TF-DNA binding in different cellular environments. Furthermore, we validate the effectiveness of the convolutional block and customized gate control by the ablation and interpretable study.

We plan to expand this article in several directions: (i) base-pair resolution TFBS prediction. Beyond discriminating the genomic region as bound versus unbound by a particular TF, it is more important to directly discover binding motifs (6–20 bp DNA sequence) from the genomic region. Recently, BPNet (Avsec et al. 2021) and U-TransNet (Zhang et al. 2022c) have been developed to perform base-pair resolution TFBS prediction for motif discovery by encoder-decoder architecture. In the future, we intend to extend the architecture of HAMPLE to utilize the encoder-decoder architecture so that HAMPLE can predict TFBS at base-pair resolution; and (ii) single-cell TF-DNA binding analysis. HAMPLE successfully performs cell-population-level TF-DNA binding prediction but ignores the inherent heterogeneity within cellular populations. Therefore, correlating single-cell sequencing data and the binding activity of TFs in individual cells is a promising direction to enable a better understanding of cellular dynamics and regulations.

## Supplementary Material

btad299_Supplementary_Data

## Data Availability

The data underlying this article are available at https://github.com/ZhangLab312/Hample/sample.
